# 

**Published:** 1844-05

**Authors:** 


### WESTERN JOURNAL

OF

### MEDICINE AND SURGERY.

### EDITORS.

---

DANIEL DRAKE, M. D.,

PROFESSOR OF PATHOLOGY AND THE PRACTICE OF MEDICINE IN THE MEDICAL INSTITUTE OF LOUISVILLE:

LUNSFORD P. YANDELL, M. D.,

PROFESSOR OF CHEMISTRY AND PHARMACY IN THE SAME:

THOMAS W. COLESCOTT, M. D.

### BOOKS AND COMMUNICATIONS RECEIVED.

We are indebted to the publishers for a copy of Stokes' valuable work on the Chest, which will receive our earliest notice. From the Medical Society of N. Y. we have received a copy of their Transactions containing several interesting papers, of which we shall have something to say hereafter. We are obliged to Dr. Bell for a copy of Nunnelly's elaborate monograph on Erysipelas, published in his Select Medical Library. Dr. Dowler, of New Orleans, has favored us with a learned communication on *Post-Mortem* Fever, which will appear in our June number.

### RECEIPTS OF THE MEDICAL JOURNAL FOR APRIL.

| | |
|---|---|
| O. Knox, Waynesboro, Tenn., - - - - | $5 00 |
| R. Steele, Paris, Ill., - - - - - | 5 00 |
| W. M. Stansbury, Carrollton, Miss., - - - | 5 00 |
| W. Nance, Vermont, Ill.. - - - - - | 5 00 |
| A. R. Kirkpatrick, Woodville, Miss., - - - | 5 00 |
| A. B. Palmer, Harrisonville, Mo., - - - - | 5 00 |
| Dr. J. A. Young, Monmouth, Ill., - - - - | 5 00 |
| Dr. Tompkins, Milroy, Ia., - - - - | 5 00 |
| Drs. Knox & Gregory, New Market, Mo., - - | 5 12 |
| B. F. Shumard, Hodgenville, Ky., - - - | 10 00 |
| J. A. Brown, Warsaw, Mo., - - - - | 5 00 |
| J. T. Daniel, Columbia, Tenn., - - - - | 15 00 |
| J. Burdett, Stanford, Ky., - - - - - | 5 00 |
| J. M. Riley, Orleans, Ia., - - - - - | 5 00 |
| P. Capshaw, Whitesburg, Ala., - - - - | 10 00 |

### TO OUR SUBSCRIBERS.

We must again remind our subscribers of their arrearages to us, and *urge* them to remit their respective dues. Our expenses for the last and present volume have been more than usually heavy, whilst the receipts, we are sorry to say, have by no means augmented in like proportion. Those who owe for this Journal from the commencement, will not, we trust, suffer us to ask them again for the amounts due us. We hope, moreover, that *all* our subscribers, whether old or recent, will feel it incumbent to remit immediately.

Thus far, we have not collected enough to defray the cost of the Journal, but we are determined to sustain it. We design commencing the ninth volume, with new and improved materials—will not the subscribers encourage and sustain us by remitting forthwith by mail, at our risk, franked by the Postmaster?

*October*, 1843. PRENTICE & WEISSINGER.

The Western Journal of Medicine and Surgery is published monthly by the undersigned, at the corner of Main and Fifth streets, Louisville, at $5 per annum, payable in advance. Each number contains 96 pages, making two volumes in the year of more than 550 pages.

Contributions to its pages by the Physicians of the Valley of the Mississippi, addressed to the Editors, are respectfully solicited.

Letters on business, to be addressed, postage paid, to the publishers.

☞Postmasters, by the regulations of the Postoffice Department, will frank letters containing subscription money, and all remittances so franked are at the risk of the publishers.

*January*, 1844. PRENTICE & WEISSINGER.

